# Protein-based vehicles for biomimetic RNAi delivery

**DOI:** 10.1186/s13036-018-0130-7

**Published:** 2019-02-26

**Authors:** Alex Eli Pottash, Christopher Kuffner, Madeleine Noonan-Shueh, Steven M. Jay

**Affiliations:** 1 Fischell Department of Bioengineering, University of Maryland, College Park, MD 20742 USA; 2 Marlene and Stewart Greenebaum Comprehensive Cancer Center, University of Maryland School of Medicine, Baltimore, MD 21201 USA; 3 Program in Molecular and Cellular Biology, University of Maryland, College Park, MD 20742 USA

**Keywords:** RNAi, Drug delivery, Protein engineering, Lipoprotein, Argonaute, Arc

## Abstract

Broad translational success of RNA interference (RNAi) technology depends on the development of effective delivery approaches. To that end, researchers have developed a variety of strategies, including chemical modification of RNA, viral and non-viral transfection approaches, and incorporation with delivery vehicles such as polymer- and lipid-based nanoparticles, engineered and native proteins, extracellular vesicles (EVs), and others. Among these, EVs and protein-based vehicles stand out as biomimetically-inspired approaches, as both proteins (e.g. Apolipoprotein A-1, Argonaute 2, and Arc) and EVs mediate intercellular RNA transfer physiologically. Proteins specifically offer significant therapeutic potential due to their biophysical and biochemical properties as well as their ability to facilitate and tolerate manipulation; these characteristics have made proteins highly successful translational therapeutic molecules in the last two decades. This review covers engineered protein vehicles for RNAi delivery along with what is currently known about naturally-occurring extracellular RNA carriers towards uncovering design rules that will inform future engineering of protein-based vehicles.

## Background

RNA interference (RNAi) is a well-studied biological phenomenon that is still emerging as a therapeutic technology. Discovered by Fire and Mello in 1998, RNAi describes the silencing of specific protein translation based on mRNA sequence complementarity of small (~ 19–23 nt) RNAs such as endogenous microRNA (miRNA) or exogenous small interfering RNA (siRNA) or small hairpin RNA (shRNA) [1]. RNAi has potentially far-reaching therapeutic potential due to the central role of aberrant protein expression in many diseases. Thus far, however, only one RNAi pharmaceutical, patisiran, has been approved for clinical use. The major obstacle to further RNAi translational successes is small RNA delivery to the cytoplasm of specific cells of therapeutic interest.

**Fig. 1:**
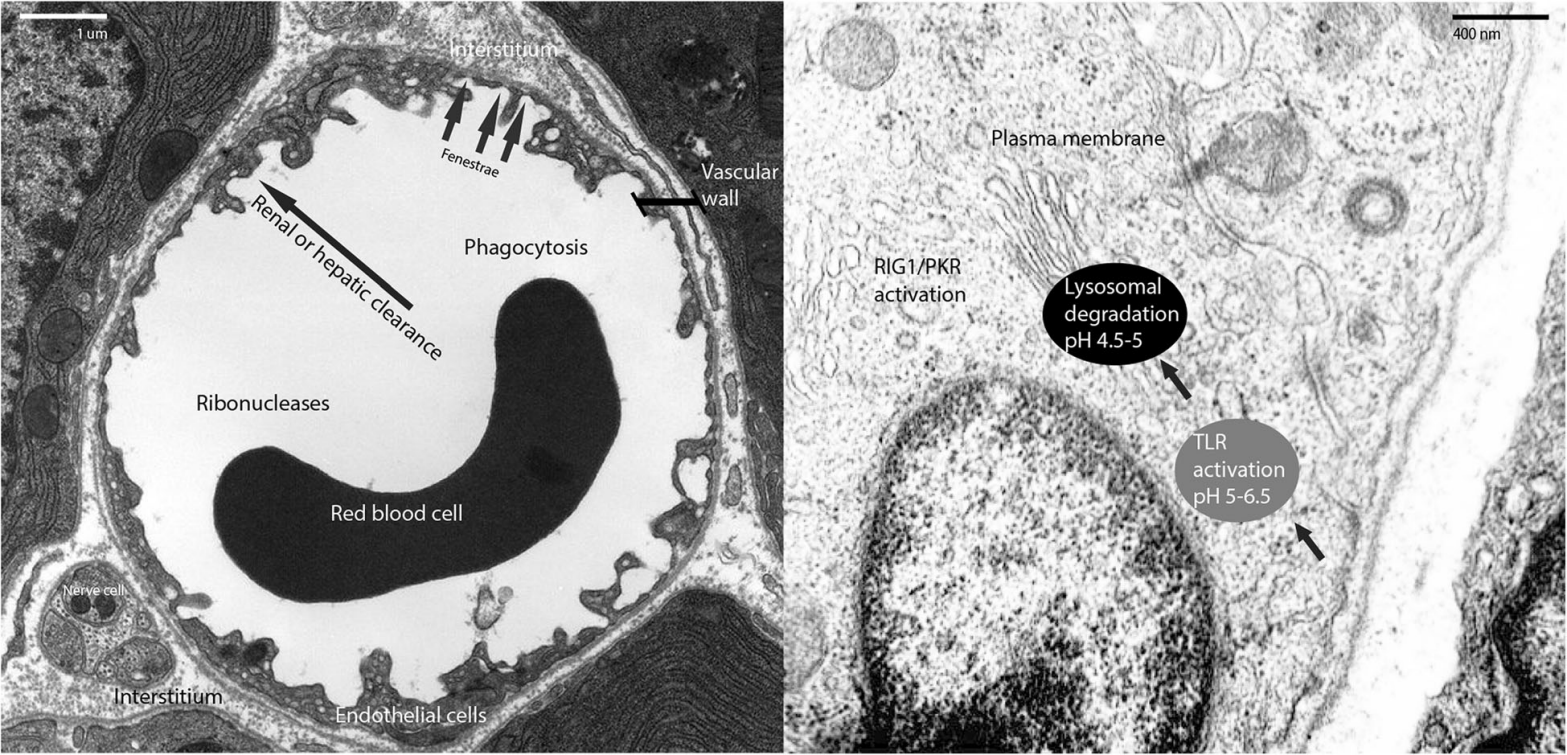
Barriers to RNA delivery. Left: RNA in circulation is vulnerable to RNase degradation and phagocytosis, and access to targeted tissue is blocked by physical barriers (e.g. endothelial and epithelial layers) and renal and hepatic clearance. Right: Cytoplasmic delivery is impaired by the plasma membrane, degradation within lysosomes, and nonspecific dsRNA immune activation. The latter can occur within the endosome by activating a Toll-like receptor (TLR) or in the cytoplasm by activating RIG1 or Protein kinase R (PKR). Images courtesy of Louisa Howard at Dartmouth University

The human body has evolved to prevent the unregulated transport of genetic material as a matter of survival. As a result, numerous biological barriers to RNAi delivery exist (Fig. 1), including: a) extracellular RNA-digesting enzymes, b) cellular membranes that repulse charged macromolecules, c) circulating phagocytic cells, d) clearance by the liver and kidneys, and e) intracellular degradation in the lysosome. These barriers have necessitated design of RNAi delivery strategies, including, prevalently, vehicles such as lipid nanoparticles and polymer-based systems. Such approaches have been shown to be effective for delivery to the liver, but can exhibit immunogenicity and be cleared by the reticuloendothelial system.

Alternatively, protein-based RNAi delivery offers a biomimetic strategy with the potential to overcome some of the obstacles that hinder synthetic systems for RNAi therapy. While RNA is trafficked within viruses and extracellular vesicles (EVs), most naturally occurring RNA transport is protein-associated or protein-mediated. Key players include apolipoprotein A-1 (ApoA1) – which constitutes the primary protein component of high-density lipoprotein (HDL) – as well as argonaute 2 (Ago2), activity-regulated cytoskeleton-associated protein (Arc), and possibly others. Leveraging biological phenomena involving proteins has already proven to be a successful formula for therapeutic development as evidenced by the clinical success of monoclonal antibodies and insulin analogs, among many others. In this review, we summarize the field of protein-based RNAi delivery, including the contribution of protein engineering approaches, and discuss what challenges and horizons remain for this biomimetic approach towards unlocking the full therapeutic potential of RNAi.

## Protein-mediated extracellular RNA transport

**Fig. 2:**
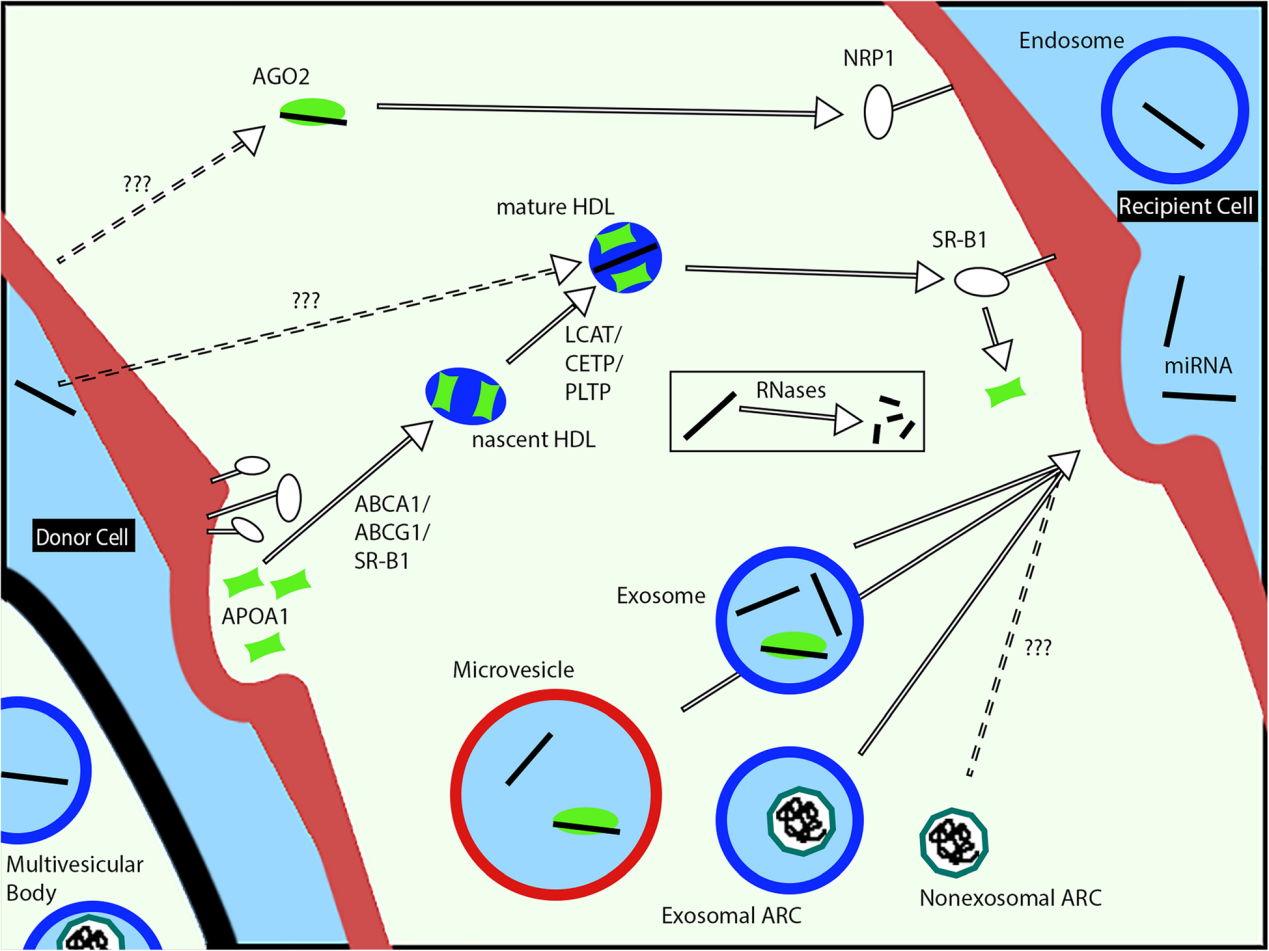
Overview of native extracellular RNA (exRNA) carriers. Unprotected RNAs are rapidly degraded in the extracellular space. Argonaute 2 (Ago2)-miRNA is found in circulation but its secretion mechanism is unknown, and it delivers via the receptor Neuropilin 1 (Nrp1). Apolipoprotein A-1 (ApoA1) is secreted by mainly the liver and intestine, and interacts with ABCA1, ABCG1, and SR-B1 in peripheral tissue to accumulate cholesterol and phospholipids. Discoidal nascent High Density Lipoprotein (HDL) is matured into spherical HDL through LCAT, PLTP, and CETP. Mature HDL is loaded with miRNA through an unknown mechanism. Nascent and mature HDL can interact with SR-B1 to deliver RNA and lipids, and lipid-free ApoA1 is released back into circulation. Spontaneous lipid transfers also play a large role in HDL function. EVs such as exosomes and microvesicles deliver RNA, as well as Ago2-miRNA and the retroviral Gag-like protein Arc. Arc has been found to mediate mRNA transport in the brain; non-exosomal Arc retains function but its prevalence is unknown. ABCA1, ATP-binding cassette subfamily A member 1; ABCG1, ATP-binding cassette subfamily G member 1; SR-B1, scavenger receptor class B type 1; LCAT, lecithin–cholesterol acyltransferase; CETP, cholesteryl ester transfer protein; PLTP, cholesteryl ester transfer protein; ARC, activity-regulated cytoskeleton-associated protein

The critical regulatory roles of small and long-noncoding RNAs are now well recognized [2, 3], however the concept of controlled extracellular RNA (exRNA) transport is more nascent. Figure 2 shows some of the most well characterized (to date) exRNA transporters, including EVs such as exosomes and microvesicles, and protein carries such as Ago2, ApoA1, and Arc. In this section, we denote the highlights of knowledge on these carriers with a focus on how such information might instruct design of biomimetic RNAi delivery strategies.

### Lipoproteins

High density lipoprotein (HDL) is a heterogeneous, complex circulating particle consisting of mainly phospholipids, cholesterol and proteins, with the primary protein component (> 70%) being apolipoprotein A-1 (ApoA1). Much has been described about the role of HDL in cholesterol efflux and its effects on cardiovascular function, but appreciation of the importance of HDL in exRNA transport is more recent. In 2011, *Vickers* et al. reported that miRNA is found in complex with HDL and showed that the HDL-associated miRNA in healthy and atherosclerotic patients differed. HDL was further found to accept miRNA from macrophage cell line J774 in vitro, with subsequent capability to deliver miRNA to hepatoma cell line Huh7 via scavenger receptor class B type 1 (SR-B1) [4]. *Wagner* et al. reported that HDL facilitated transport of low levels (5–10 copies/cell) of miRs to endothelial cells in vitro [5]. *Tabet* et al. showed that native HDL delivered high levels of miR-223, a downregulator of intercellular adhesion molecule-1 (ICAM-1) mRNA, resulting in ICAM-1 knockdown in endothelial cells in vitro [6]. Additionally, many studies have examined Low Density Lipoprotein (LDL) association with miRNA, with the consensus being that levels of miRNA associated with LDL are much lower than HDL [7]. Meanwhile, a recent study has observed that a significant amount of lipoprotein-RNA is non-host derived [8].

HDL delivers cargo via at least one known receptor, SR-B1, which is widely expressed in macrophages as well as in tissues such as fat, endothelium, intestines, and brain (HDL can cross the blood-brain barrier) [9]. The highest expression occurs in the liver and steroidogenic tissues that utilize cholesterol for bile and hormone synthesis, respectively [10]. Expression is also high in many tumors [11]. SR-B1 binds to HDL and forms a non-aqueous channel between the lipoprotein and the plasma membrane, through which lipophilic molecules can travel bidirectionally (down a concentration gradient) [10]. Therefore, HDL achieves a direct cytoplasmic delivery. Controversially, there have been reports that SR-B1 also mediates HDL endocytosis and resecretion, potentially playing a role in non-lipid delivery. In hepatocytes, HDL is resecreted deplete of cholesterol, while in macrophages, HDL is resecreted replete with cholesterol, indicating that cell type and cholesterol level play a role in HDL function [12].

There are still open questions as to how miRNA is taken up, is taken up, bound to, and delivered by HDL, what the true axis of communication is, and the role of non-host organism-derived RNA.

### Argonaute 2

Argonaute 2 (Ago2) is the catalytic center of the RNA-Induced Silencing Complex (RISC) that accepts miRNA and siRNA, protects it from degradation, and cleaves complementary mRNA in the cytoplasm. Ago2 has been well-studied within the cell, but in 2011, *Arroyo* et al. and *Turchinovich* et al. reported that a majority of miRNA in circulation was not associated with vesicles, but rather protein – specifically ~ 100 kDa Ago2 [13, 14]. The distribution of miRNA among the two fractions was uneven, indicating a sorting mechanism. *Arroyo* et al. estimated that potentially 90% of extracellular miRNA were Ago2-bound. A 2016 paper from *Prud’homme* et al. identified Neuropilin-1 (Nrp1) as a receptor for extracellular Ago2, and demonstrated functionalized delivery in multiple cell lines [15]. Nrp1 is also a receptor for VEGF and Semaphorin 3, among others, and is expressed widely in endothelial, immune, and many cancer cells, as well as others, including in the developing brain and heart [16, 17]. The results above suggest a major intercellular communication system based on protein-mediated miRNA delivery. This communication system would be privileged; endogenous miRNA must compete for Ago2 loading, but exogenous miRNA would be pre-loaded and ready to perform. However, there are currently more questions surrounding extracellular Ago2 than answers. Ago2 secretion mechanisms are currently unknown, though may be related to one of many binding partners, such as Hsp90 or Hsc70 [18]. It is also unknown if Ago2 has any mechanism for targeting specific tissues.

### Arc protein

Activity-Regulated Cytoskeleton-Associated protein (Arc) is a major regulator involved in synaptic plasticity and maturation, learning, and memory [19]. Arc is an early immediate neuronal gene that regulates synaptic plasticity through AMPA receptors, which are involved in rapid synaptic transmission. Arc mRNA moves to the dendritic spines where it is locally translated and begins engaging with the endocytic machinery to regulate the AMPA receptors [20]. Regulation of Arc expression is essential for normal cognition and long-term memory storage. Abnormal Arc expression has been implicated in various neurological and neurodevelopmental disorders such as Alzheimer’s disease, Angelman syndrome, Fragile X syndrome, and schizophrenia [19]. Previous studies have noted the similarity between viral proteins and Arc, as it is composed of structural elements also found in Group-specific antigen (Gag) polyproteins encoded in retroviruses and retrotransposons, including human immunodeficiency virus type 1 (HIV-1) [21].

In 2018, *Pastuzyn* et al. and *Ashley* et al. reported a novel mechanism by which genetic information (mRNA) is transferred between neurons via Arc [21, 22]. Arc encapsulates mRNA into viral-like capsids for delivery to neighboring neurons within EVs. When purified in bacterial systems, Arc spontaneously self assembles into oligomeric structures with biochemical properties similar to Gag proteins. Arc capsids are double-shelled structures measuring 32 nm in diameter and are capable of binding RNA nonspecifically, which was found to be a requisite for normal capsid formation. It is hypothesized that Arc is co-expressed with, and encapsulates and delivers, Arc mRNA, which may constitute a positive feedback system of Arc expression. Arc proteins are secreted within EVs, the uptake of which is thought to be dictated by targeting moieties on the lipid surface while the capsid itself protects and transfers the mRNA. It was also shown that Arc capsids delivered functional mRNA even without EV encapsulation [21]. Further investigation of this unprecedented viral-like mechanism may elucidate understanding of developments of neurological disorders. Additional tests must be performed to determine if mRNA binding truly is nonspecific and if non-encapsulated Arc is truly functional and non-immunogenic. Finally, the symbiotic relationship between “human” and “viral” DNA displayed here raises existential questions about genetic identity.

### Non-protein vehicles

While this review does not focus on pathogenic RNA vectors, other physiologic vectors have significant roles in exRNA transport, namely EVs. The discovery by *Valadi* et al. that EVs (including exosomes, microvesicles, and apoptotic bodies) transfer RNA species opened a new frontier of knowledge on intercellular communication [23]. Previously, EVs, which are secreted by most –if not all– cell types and are prevalent in all body fluids, were considered a form of cellular waste disposal. Since *Valadi* et al., an entire field of studying the natural pathways of EV biogenesis, composition, and function has emerged. While much is still unknown about these heterogeneous vesicles, it is clear that their RNA transfer capability plays an important role in healthy physiology as well as pathologic progression. It has also been reported that some EVs may have the ability to target specific cell types based on their surface proteins [24]. EVs can also enhance their signaling power by co-delivering co-factors for RNAi function, such as Ago2 [25]. Further detail on this topic is outside the scope of this review, however the reader is referred to excellent recent review articles for additional information [26, 27].

## Engineering of protein-mediated RNA delivery

**Fig. 3:**
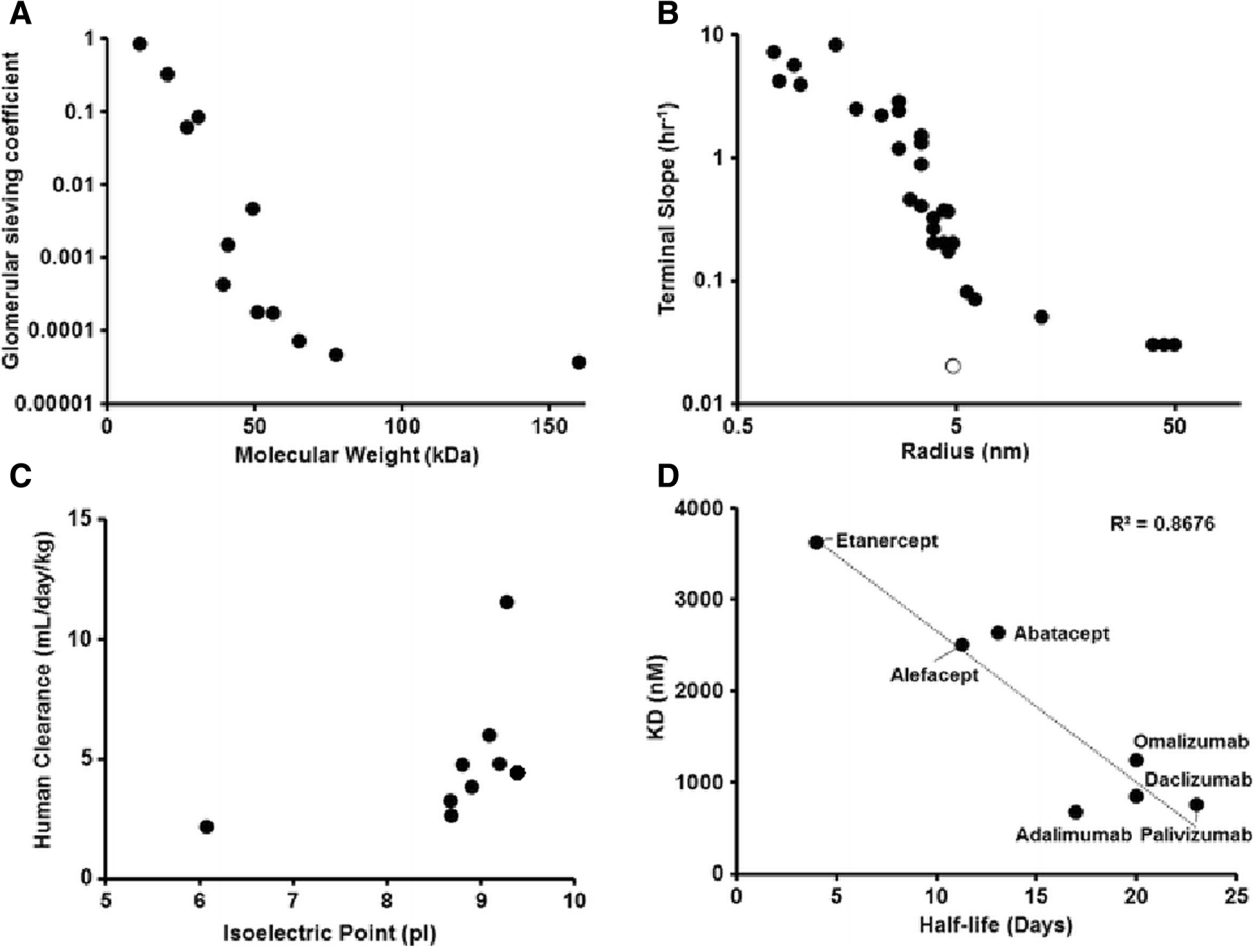
Trends in pharmacokinetic behavior of therapeutic proteins. **a** Glomerular sieving coefficient, which is representative of renal clearance, is inversely related to molecular weight, so smaller molecules are excreted faster. **b** Terminal slope of pharmacokinetic profile, which corresponds to rate of systemic clearance from the body, is inversely related to hydrodynamic radius, so smaller molecules are cleared faster (open dot represents IgG which utilizes FcRn pathway). **c** Systemic clearance is related to molecular charge, so more negative molecules are cleared faster (higher pI corresponds to more negative charge). **d** Half-life is related to binding affinity, so molecules with worse affinity are cleared faster. Reprinted with permission pending from [30]. Reproduced with permission from Springer Nature, Journal of Pharmacokinetics and Pharmacodynamics (Pharmacokinetic and pharmacodynamic considerations for the next generation protein therapeutics, Dhaval K. Shah, copyright (2015)

A feature of protein-based therapeutic systems is manipulability, or “engineerability.” Many molecular attributes that contribute to optimal pharmacologic efficacy – such as low immunogenicity, avoidance of renal and other forms of clearance, and prevention of opsonization-mediated phagocytosis and degradation (Fig. 3) – can be incorporated into proteins via straightforward genetic engineering techniques. Protein size, charge, post-translational modification, and binding affinity to both cargo (e.g. RNA) and target moieties can all be manipulated using rational design or directed evolution approaches. For example, conjugation of a therapeutic protein to the Fc domain or albumin-binding domain can markedly extend its half-life [28]. These same domains, along with a variety of others, could also be appended to increase protein size, an important determinant of molecular pharmacokinetics. Molecules greater than 60 kDa avoid renal clearance, while molecular weight is inversely related to endothelial permeability and tissue penetrance (and smaller molecules are more highly influenced by target binding affinity) [29, 30].

With regard to optimizing RNAi delivery, protein-based vehicles (and all vehicles in general) must accomplish protection of the RNA strand, evasion of clearance, cell/tissue targeting, cell penetration, and RNAi lysosomal escape. Theoretically, this could result in a Rube Goldberg-esque chimera containing: a) an RNA-binding domain, b) a tissue-targeting domain, c) an endocytic domain, d) an endosomolytic domain (this is often added as a second agent), e) a half-life enhancing domain, and f) multiple flexible linkers. Unfortunately, any such vehicle would likely have low translational potential due to its complexity. Thus, attempts at engineering protein vehicles for small RNA delivery to date have in most cases focused on more practical approaches, including leveraging biomimicry. Here, we present a summary of the progress in the field, organized by vehicle RNA-binding domain.

### High density lipoprotein

Due to its size, long half-life (5.8 days for ApoA1), anti-inflammatory nature, and low toxicity, HDL has recently received attention as drug delivery vehicle, mainly targeting the liver or tumors [31–33]. Additionally, the amphipathic nature of HDL allows for loading of hydrophobic, hydrophilic, or amphipathic molecules. HDL can be isolated from native blood samples (nHDL) or reconstituted in vitro with recombinant ApoA1 (rHDL), most commonly with a cholate method [34]. Reconstitution has multiple advantages, such as availability and low risk of contamination, and depending on the lipids used, rHDL can mimic nHDL at any stage of maturity.

Long before the discovery of miRNA-HDL complexes in blood, molecular engineers had experimented with cholesterol-conjugated siRNA and antisense oligonucleotides [35–37]. Especially of note is the knockdown of apolipoprotein B (ApoB) in non-human primates via chol-siRNA injection in 2006 by *Zimmermann* et al [38]*.* In 2007, researchers associated with Alnylam Pharmaceuticals published a wide-ranging study of various lipophilic siRNA conjugates and their relative efficacy in murine hepatic delivery [39]. They showed that the lipophile-siRNAs that preferentially associated with HDL rather than albumin (or remained unbound) were most effective in knocking down the target (ApoB) mRNA in the liver. Strikingly, pre-incubating cholesterol-siRNA (chol-siRNA) with native HDL before injection led to ~ 2- to 4-fold less plasma ApoB (produced in the liver) when compared to chol-siRNA injected alone. The authors examined the biodistribution of chol-siRNA, with greatest uptake in liver, kidney, adrenal, and ovary tissues. They also demonstrated that HDL-mediated delivery depends on SR-B1 and, interestingly, lipophilic-siRNA delivery depends on SidT1, a mammalian homologue to the Sid1 transmembrane protein that regulates systemic RNA transport in *C. elegans.* In 2012, another group associated with Alnylam, *Nakayama* et al.*,* compared the liver delivery of chol-siRNA reconstituted with either recombinant ApoA1 or apolipoprotein E (ApoE) [40]. ApoE primarily binds to the LDL Receptor (LDLR), which may have led to greater liver delivery, and therefore siRNA efficacy, of ApoE-rHDL over ApoA1-rHDL. The authors also saw that adding 4 chol-siRNA molecules for every 1 rHDL (of either type) led to siRNA buildup on the plasma membrane in vitro, as opposed to cytoplasmic buildup seen with 1:1 loading. This indicates that there may be a limit to how much siRNA can be loaded using this cholesterol-conjugated method before it interferes with receptor binding. A possible solution to this problem was introduced by *Shahzad* et al., who applied a different strategy for delivery of non-cholesterol-conjugated siRNA; they loaded anionic siRNA into the core of rHDL by neutralizing with cationic oligolysine peptides [11]. This approach may increase the siRNA loading capacity of rHDL. The group used siRNA against STAT3 and FAK in mouse models of ovarian and colorectal cancer, alone or in combination with chemotherapeutics. Results showed that in three different models, including a resistance model, STAT3-rHDL monotherapy or in combination with docetaxel or oxaliplatin averaged ~ 72% and ~ 93% decrease in tumor weight, respectively. Liver function was not impacted and empty rHDL did not affect tumor weight. Additionally, the authors reported that siRNA was distributed evenly to 80% of a given tumor after injection. An analysis by *Ding* et al.*,* which utilized ApoA1-incorporated liposomes at a diameter of ~ 90 nm, nevertheless showed that SR-B1-mediated chol-siRNA uptake is similar to cholesteryl ester selective uptake [41]. Alternatively, some groups have utilized ApoA1 mimetic peptides [42–44], gold-templated nanoparticles [45, 46] and ApoA1-incorporated liposomes [41, 47–50] to deliver siRNA. This review will not cover those strategies in detail.

There have been relevant attempts to further engineer the HDL molecule for enhanced drug delivery. Some groups have sought to enhance targeting capabilities by incorporating targeting moieties to HDL to help direct delivery to the liver [51] or tumor [52]. Some groups have encapsulated various packages within the core, such as super paramagnetic nanoparticles for guided targeting [53], or hydrophobic chemotherapeutics [54–56] and Vitamin E [57] for cancer therapy. Any incorporation or encapsulation method may increase the size of the rHDL molecule, which could impact delivery. Additionally, naturally occurring variants of ApoA1, including the Milano and Paris mutants, have been discovered. These variants, R173C and R151C mutants, respectively, perform greater cholesterol efflux due to more transient cholesterol binding [58, 59]. Their behavior in a system of siRNA delivery is currently unknown.

### Albumin

Human serum albumin (HSA) is the most abundant protein in blood. It is distributed throughout the blood circulation and has exceptionally low immunogenicity and long half-life [60]. Previous success in harnessing HSA as a drug delivery vehicle makes it attractive for RNA delivery. HSA, like RNA, is a negatively charged molecule and the two do not spontaneously interact. However, *Sarett* et al. showed that lipophilic DSPE-PEG-conjugated siRNA was capable of binding endogenous HSA [61]. In a mouse model, HSA-binding reduced renal clearance and improved half-life of modified siRNA, and enhanced delivery to the tumor, achieving a tumor:liver delivery ratio over 40 (in comparison to ~ 3 for jetPEI, a cationic polymer). Others have modified the charge of the albumin to generate electrostatic attraction with RNA. *Han* et al. modified the isoelectric point of bovine albumin with ethylenediamine, making it positively charged at the pH of blood and able to spontaneously form complexes with negative RNA [62]. In mice, these molecules were distributed primarily to the lungs (5–12:1 lung:liver delivery ratio) and reduced the number of lung cancer metastases by over half. *Wen* et al. made RNA-HSA complexes by mixing unmodified molecules at pH 4, at which HSA is positively charged. Thermal treatment crosslinked the complexes, which remained stable at blood pH [63].

### p19

The p19 protein of the Tombusvirus genus has been developed as a siRNA delivery vehicle by a number of groups, but has not shown success in any in vivo environments. Originally detected as function-ambiguous subgenomic RNA in the tomato bushy stunt plant (and named for its size), the 19 kiloDalton (kDa) protein was found to greatly enhance systemic invasion of plants [64–67]. *Voinnet* et al. showed that p19 was a viral counter-defense to posttranscriptional gene silencing (PTGS), the analogue of RNAi in the plant kingdom [68]. Further studies elucidated that p19 dimers selectively bind to small double-stranded RNA (dsRNA) ~ 19–21 bp in length with subnanomolar affinity, behaving as a “molecular caliper” [69–71]. Engineering of the p19 protein began with *Cheng* et al. enhancing dsRNA affinity by linking two p19 monomers [72]. *Choi* et al. fused the ephrin mimetic peptide YSA to p19 monomers to effectively target siRNA to EphA2-expressing cancer cells in vitro [73]. This group saw a ~ 6- to 36-fold extension of siRNA half-life in 30% serum when first incubated with p19-YSA. Additionally, they saw protein-RNA dissociation at endosomal pH. *Danielson* et al. fused a cell-penetrating Tat peptide to p19 dimers, and saw substantial knockdown in vitro only when co-treated with cell-penetrating endosomolytic compound E5-TAT [74]. *Yang* et al. performed yeast-display directed evolution on p19, ultimately finding a double mutant with 160-fold greater binding affinity [75]. The p19 monomers were then fused to an EGFR-targeting domain and added to cells in vitro, along with an EGFR-targeting endosomolytic compound. Experiments showed that higher affinity led to greater silencing efficacy. The authors attributed this to increased uptake as well as enhanced intracellular pharmacodynamics.

### Antibodies

Some designs have utilized antibodies as targeting moieties for specific delivery, but others have conjugated RNA directly to antibodies themselves [76]. *Cuellar* et al. utilized THIOMAB antibodies covalently bound to siRNA to form antibody-siRNA conjugates [77]. These antibodies are referred to as THIOMABs since they contained an exposed cysteine residue on each heavy chain to which the cargo was attached, allowing for production of homogeneous antibody-drug conjugates [78]. These constructs targeted tumor cells in mice, but were limited by endosomal entrapment and intracellular clearance. *Xia* et al. used streptavidin-conjugated antibodies and biotinylated siRNA to deliver in vitro, but also saw issues with endosomal degradation [79]. *Sugo* et al. conjugated thiol-reactive siRNA to a single-chain variable fragment (scFv) antibody for CD71 in order to deliver to mouse heart and skeletal muscle [80]. Remarkably, they observed persistent knockdown (30 and 62%, respectively) even one month later.

### PKR

Protein Kinase R (PKR) is an interferon-induced kinase that is a key component in the antiviral innate immune pathway in eukaryotes. PKR is activated by double stranded viral RNAs, a byproduct of transcription in RNA/DNA viruses. Once activated, PKR phosphorylates eukaryotic initiation factor-2, which inhibits translation of viral proteins and subsequent viral spread.

PKR is one of the well-studied proteins with canonical dsRNA binding motifs. The protein contains two dsRNA binding domains (DRBD), one at the N- terminus and one at the C-terminus connected by a long linker [81]. The DRBDs consist of two tandem binding motifs, dsRBM1 and dsRBM2 joined by a 20-residue linker to form the αβββα fold. It is thought that dsRNA binds to PKR in a sequence independent manner. The crystal structure shows the protein spanning 16 bp of the dsRNA and primarily interacting with 2′-hydroxyls and the phosphate backbone of the dsRNA [82].

*Eguchi* et al. developed the fusion protein PTD-DRBD, now commercially known as Transductin, comprised of the PKR binding domains and a Tat peptide that showed effective siRNA delivery in various cell lines. However, in vivo studies showed an observed non-specific cell uptake, which caused several side effects [83]. It was therefore thought that replacing the Tat sequence with a receptor ligand would allow for specific targeting. *Geoghegan* et al. replaced the Tat peptide with B2 peptide sequence that binds to a recombinant transferrin receptor. The fusion protein was shown to effectively knockdown HPRT in HeLa cells and showed TfR mediated uptake. It was also noted that knockdown was enhanced with chloroquine suggesting the endosomal entrapment of the complexed protein [84]. In 2014, *Lui* et al. developed a multiagent siRNA delivery system consisting of the dsRBD domain, an EGFR clustering domain, and a pore-forming protein Perfringolysin O (PFO) domain to induce endosomal escape. The delivery system showed efficient silencing in vitro but did not achieve delivery in vivo due to the dissociation of the siRNA from the protein [85].

### Viral vectors and virus-like particles

~ 70% of gene therapy clinical trials have utilized modified viruses, starting in 1989, before the discovery of RNAi [86]. Some viruses deliver genetic material for transient expression, while others integrate into the genome, allowing for long-term expression. Long-term expression is usually preferred, though when coupled with broad tropism (which many viruses exhibit) can be dangerous [87]. Additionally, genome integration can be carcinogenic [88]. Furthermore, in one case, extended genomic expression of exogenous shRNA in the liver consistently led to fatality in mice due to saturation of RNAi machinery [89]. Other concerns that have cooled interest in viral delivery are potential immunogenicity, viral sequence mutation, and difficulty in large-scale manufacture [90, 91]. However, there are also advantages to using viral vectors. Viruses have been evolutionarily honed for delivery to the mammalian cell cytoplasm (and nucleus), and they do so extremely efficiently and in low doses. Additionally, viruses have recently been approved by the FDA for multiple diseases: the treatment of inoperable melanoma, as an ocular gene delivery vehicle for hereditary retinal dystrophy, and for the transfection of chimeric antigen receptor T-cells. There are many reviews that focus on viral vectors for gene delivery [92–94]. There have been strategies to improve viral molecules for targeting, including pseudotyping and introducing adaptor and binding domains [87, 95]. Other attempts to optimize viral vectors as drug delivery vehicles are ongoing as well [96, 97].

Heterologous expression of the major structural proteins of viruses leads to the self-assembly of virus-like particles (VLPs). VLPs have similar structural formation of the parental virus without any secondary proteins or genomic data, and thereby forego some of the concerns with viral delivery discussed above. Unlike viruses, VLPs can be produced in high-yield expression systems such as *E. coli* or insect cells and are more easily manipulable. All VLPs discussed here are ~ 24–40 nm in diameter. A common strategy available with some VLPs is encapsulation of cargo via disassembly-reassembly, whereby reduction of disulfide bonds leads to VLP dissociation and dialysis into a oxidizing environment in the presence of nucleic acids leads to packaging [98]. *Bousarghin* et al. utilized this strategy with a VLP based on human papillomavirus virus (HPV) capsid protein L1, and encapsulated plasmid DNA that expressed shRNA [99]. This shRNA targeted p53-inhibiting proteins, and halved tumor weight in a mouse model of HPV-caused cervical cancer. The same disassembly-reassembly strategy was used in VLPs based on JC virus by two different groups [100, 101]. *Chou* et al. injected VLPs containing IL-10 shRNA into mice along with immunogenic LPS, and saw a massive reduction of IL-10 and TNF-α in the bloodstream, by 93 and 81%, respectively, and improved mouse survival. *Hoffmann* et al. performed extensive in vivo studies looking at delivery of VLP-siRNA to the tibia and lumbar vertebrae in mice. They observed up to a 40% decrease in RANKL mRNA that was dose-dependent and sustained with multiple injections.

A second strategy is to encapsulate the RNA through binding to the internal face of a capsid. Often, as in the case of the coat protein from bacteriophage MS2, the VLP will only form when stabilized by the presence of specific RNA sequences. *Ashley* et al. co-packaged four different siRNA molecules (~ 84 molecules/VLP) into MS2 VLPs, finding that a specific sequence was not required for them [102]. They also conjugated a peptide for targeting and saw a remarkable increase in endocytic specificity in vitro. *Pan* et al. packaged pre-miR-146a into MS2 VLPs using a specific sequence called a *pac* site and then conjugated a TAT peptide [103]. In mice, they saw almost equal concentration of the miRNA in plasma, lung, spleen, and kidney. *Galaway* et al. packaged siRNA into MS2 VLPs using a specific “TR” sequence, and later conjugated transferrin for targeting [104]. *Fang* et al. used a specific hairpin to load miR-30 into a VLP derived from the bacteriophage Qβ [105]. A third strategy was employed by *Choi* et al., wherein they made a chimera of truncated Hepatitus B Virus (HBV) capsid protein, RGD peptide (for targeting), and p19 (for RNA binding) [106, 107]. This construct greatly reduced tumor size in a mouse model. A fourth strategy involves nano-scale self-assembled protein structures that are not virally derived: nanocages. In work by *Lee* et al., each ferritin-based nanocage was designed to display 24 polypeptides with the following constitution: lysosome-exclusive cleavable peptide – cationic protamine-derived peptide (which associated with siRNA) – EGFR-targeting affibody – cell-penetrating Tat peptide [108]. Likewise, *Guan* et al. designed a heat shock protein-based nanocage that displayed an arginine-rich peptide for cell penetration (see below) [109].

### Naturally-occurring cationic peptides

Cationic peptides that have been used for small RNA delivery have been covered by *Shukla* et al. [110]. In general, vehicles that display a high concentration of positive charge often suffer due to high retention in all tissues, including those that are not being targeted [111, 112]. Here we briefly discuss naturally-occurring cationic peptides. Protamine is a naturally-occuring peptide with a high percentage of arginine (67%) that is FDA approved. In nature, protamine condenses DNA of fish sperm for delivery to the nucleus of an egg. This property has led to research into its potential as an siRNA carrier. In one attempt, siRNA as well as cholesterol were condensed by protamine into a nanocomplex that showed preferential endocytosis into liver cells in vitro [113, 114]. Protamine has also been fused to antibodies and antibody fragments for targeted siRNA delivery to tumors, and shown inhibition of tumor genes in mouse models [76, 115–117]. Some groups have also utilized atelocollagen, which is collagen treated with pepsin, as a small RNA delivery vehicle [118–121]. Other groups have used gelatin, another collagen derivative.

### Cell-penetrating and Endosomolytic peptides

Much focus has been directed at devising simple peptides for cytoplasmic delivery of siRNA. Cell-penetrating and endosomolytic peptides interact with the plasma membrane or the endosome membrane, respectively, in a biophysical manner in order to pass through the bilayer. These peptides are most effectively used in conjunction with targeting moieties since they are nonspecific and will interact with any cell type. This promiscuity contributes to their overall toxicity [122]. There have been a number of reviews on these peptides in the context of siRNA delivery [123–125]. Briefly, cationic arginine-rich peptides, such as the Tat peptide, interact with negatively charged phospholipids on the cell surface and can create transient pores in the membrane. Amphipathic peptides insert themselves into the lipid bilayer and can traverse the plasma membrane in this manner. These mechanisms are also related to endocytosis, however, and can lead to accumulation in the endosome [122]. Endosomolytic peptides are specifically designed to be reactive to the low pH environment. Fusogenic peptides change confirmation to become amphipathic helices which fuse to and disrupt the endosome. Some peptides have masked reactive moieties that are revealed through a pH-sensitive chemical reaction. Proton buffering peptides have weak bases and act as a proton sponge, accumulating protons and causing osmotic swelling and/or rupture. Some light-activated peptides have even been developed for endosomolytic escape. There are ongoing attempts to design peptides that exhibit both cell-penetrating and endosomolytic capabilities [126].

## Conclusions

Further knowledge development on the natural pathways of RNA communication between cells would inform novel biomimetic therapeutic RNAi delivery strategies. In the current landscape, the study of EVs in this role has eclipsed the study of other biological vehicles, however other natural vehicles are important to study if only to understand the limitations of EV-mediated transport. Important questions to ask are: 1) why have we evolved multiple miRNA transport mechanisms?; 2) are these redundant pathways?; and 3) what is the axis of communication for each of these vehicles?

Additionally, understanding the various functions of each of the natural vehicles would inform design of engineered RNAi delivery. The initial discovery by *Valadi* et al. of physiological RNA transport through EVs led to work by *Alvarez-Erviti* et al. which delivered exogenous siRNA to the mouse brain, and many further works [23, 127]. Indeed, in a few short years the EV research field has ballooned; now there are studies on both diagnostics and therapeutic delivery for a bevy of diseases. In a broader sense, however, scientists have been studying synthetic EVs for drug delivery since the 1970s in liposomes and lipid nanoparticles. The potency of lipid-based drug delivery seems obvious in retrospect; given what we now know about the natural pathways of EV-mediated delivery, we can refer to lipid systems as biomimetic.

Biomimicry is particularly effective in that it can incorporate therapeutic factors that we cannot yet design rationally. In the case of noncovalent protein-based RNAi delivery, affinity of the carrier for the RNA is an important factor for stability in circulation (and intracellularly), and scientists have sought to enhance delivery efficiency by enhancing affinity. For example, *Yang* et al. enhanced the binding affinity of p19 for dsRNA through yeast display to a dissociation constant (*k*_*d*_) of 11 pM [75]. Contrast this with the reported affinity of ~ 72 nM for Ago2 and ssRNA, and it remains unclear why Ago2 would retain stability in circulation and other constructs would not [128]. In reality, however, the process of Ago2 binding to RNA has been described as “irreversible,” and the half-life for the complex may be days or weeks in vitro [129, 130]. Is such intra-vehicular affinity required for successful delivery? It is clear we need to research specific problems in noncovalent RNAi delivery to begin to understand the role of factors like affinity.

Additionally, further research needs to be done on the pharmacokinetic and pharmacodynamic tools of analysis of protein-based RNAi treatment. While this is true of protein therapeutics in general [131], small RNA delivery poses its own unique challenges. Efficacy is dependent on efficient cytoplasmic delivery to the proper cells (followed by additional processing). The downstream effects of various small RNA are divergent by definition, but normative methods of relating small RNA to mRNA to protein levels over a given time will provide tools for devising doses and time courses and analyzing pharmacokinetic profiles for definition of a therapeutic window. Toxicology for small RNA is also very important but complex, as deleterious effects are likely sequence- and organ-specific. However, clever models like a transgenic mouse that expresses fluorescent protein in the presence of small RNA can simplify biodistribution studies [132]. The pharmacological rules governing small RNA efficacy would likely inform iterative vehicle design.

Proteins are inherently unstable and complex molecules. In production, they are subject to various unintentional processes which render them ineffective: heterogeneity, chemical and enzymatic hydrolysis, crosslinking/aggregation, side-chain modification, irreversible conformation changes, unfolding, and others. They are sensitive to pH, temperature, ionic concentration, and other formulation properties. In vivo, they are vulnerable to proteases in circulation and are highly bioactive and thus likely to produce unintended effects. The specific factors that are currently limiting protein-RNA vehicles are construct-dependent, but in general include instability in circulation, rapid clearance, inability to circumvent endosomal degradation, and nonspecific delivery. It is our hope that by increasing understanding of physiological exRNA transport and taking seriously pharmacokinetic restraints, protein-based RNAi delivery vehicles could overcome current limitations and push RNAi therapeutics further into the clinic.

## Abbreviations

Ago2: Argonaute 2
ApoA1: Apolipoprotein A-1
ApoB: Apolipoprotein B
ApoE: Apolipoprotein E
Arc: Activity-regulated cytoskeleton-associated protein
chol-siRNA: Cholesterol-siRNA
DRBD: DsRNA binding domain
EV: Extracellular vesicle
exRNA: Extracellular RNA
Gag: Group-specific antigen
HDL: High-density lipoprotein
HPV: human papillomavirus
HSA: Human serum albumin
ICAM-1: Intercellular adhesion molecule-1
kDa: KiloDalton
nHDL: Native HDL
Nrp1: Neuropilin-1
PKR: Protein kinase R
rHDL: reconstituted HDL
RISC: RNA-Induced Silencing Complex
RNAi: RNA interference
SR-B1: Scavenger receptor class B type 1
VLPs: Virus-like particles
